# Machine Learning and Pathway Analysis-Based Discovery of Metabolomic Markers Relating to Chronic Pain Phenotypes

**DOI:** 10.3390/ijms23095085

**Published:** 2022-05-03

**Authors:** Teemu Miettinen, Anni I. Nieminen, Pekka Mäntyselkä, Eija Kalso, Jörn Lötsch

**Affiliations:** 1 Pain Clinic, Department of Perioperative Medicine, Intensive Care and Pain Medicine, Helsinki University Hospital and SleepWell Research Programme, University of Helsinki, 00014 Helsinki, Finland; teemu.miettinen@helsinki.fi (T.M.); eija.kalso@helsinki.fi (E.K.); 2 Metabolomics Unit, Institute for Molecular Medicine Finland (FIMM), University of Helsinki, 00014 Helsinki, Finland; anni.nieminen@helsinki.fi; 3 Institute of Public Health and Clinical Nutrition, University of Eastern Finland, Kuopio Finland, and Primary Health Care Unit, Kuopio University Hospital, 70211 Kuopio, Finland; pekka.mantyselka@uef.fi; 4 Institute of Clinical Pharmacology, Goethe—University, Theodor—Stern—Kai 7, 60590 Frankfurt am Main, Germany; 5 Fraunhofer Institute for Translational Medicine and Pharmacology ITMP, Theodor-Stern-Kai 7, 60596 Frankfurt am Main, Germany

**Keywords:** chronic pain phenotypes, sleep disorders, obesity, metabolic markers, metabolic pathways, supervised machine

## Abstract

Recent scientific evidence suggests that chronic pain phenotypes are reflected in metabolomic changes. However, problems associated with chronic pain, such as sleep disorders or obesity, may complicate the metabolome pattern. Such a complex phenotype was investigated to identify common metabolomics markers at the interface of persistent pain, sleep, and obesity in 71 men and 122 women undergoing tertiary pain care. They were examined for patterns in d = 97 metabolomic markers that segregated patients with a relatively benign pain phenotype (low and little bothersome pain) from those with more severe clinical symptoms (high pain intensity, more bothersome pain, and co-occurring problems such as sleep disturbance). Two independent lines of data analysis were pursued. First, a data-driven supervised machine learning-based approach was used to identify the most informative metabolic markers for complex phenotype assignment. This pointed primarily at adenosine monophosphate (AMP), asparagine, deoxycytidine, glucuronic acid, and propionylcarnitine, and secondarily at cysteine and nicotinamide adenine dinucleotide (NAD) as informative for assigning patients to clinical pain phenotypes. After this, a hypothesis-driven analysis of metabolic pathways was performed, including sleep and obesity. In both the first and second line of analysis, three metabolic markers (NAD, AMP, and cysteine) were found to be relevant, including metabolic pathway analysis in obesity, associated with changes in amino acid metabolism, and sleep problems, associated with downregulated methionine metabolism. Taken together, present findings provide evidence that metabolomic changes associated with co-occurring problems may play a role in the development of severe pain. Co-occurring problems may influence each other at the metabolomic level. Because the methionine and glutathione metabolic pathways are physiologically linked, sleep problems appear to be associated with the first metabolic pathway, whereas obesity may be associated with the second.

## 1. Introduction

Recent advances in knowledge of the biochemical basis of the pathophysiological processes involved in pain have involved metabolic processes in the production or degradation of active endogenous or exogenous molecules relevant to pain modulation [1]. Metabolic markers have been associated with various pain etiologies, such as rheumatoid arthritis [2,3], interstitial cystitis [4], or fibromyalgia [5,6]. For example, processes related to energy metabolism or biochemical changes in lipids and amino acids have been shown to differ in fibromyalgia patients from healthy controls [7]. In addition, metabolomic markers have been associated with specific clinical presentations of pain, examples being choline, phosphocholine, alanine, and taurine levels with the presence of nociceptive or neuropathic pain [8], ornithine levels with the characteristics of musculoskeletal pain [9], or epiandrosterone sulfate levels with widespread pain [10,11]. Glutamate levels may also be associated with nociception for various pain conditions [12].

The interplay between pain and metabolomics is likely complex, as persistent pain is often accompanied by a host of co-occurring problems which may also manifest at the metabolomics level. In a recent study of a cohort of 277 patients undergoing tertiary care for persistent pain, six different pain phenotype parameters yielded a subgroup structure based primarily on affective pain interference and number of pain areas [13]. Interestingly, among the 54 non-pain-related parameters, sleep problems proved most relevant for assigning a patient to the pain phenotype subgroup. A high number of chronic pain patients suffer from sleep problems. The prevalence for insomnia was 39.8% among those with fibromyalgia and 25.1% among those with musculoskeletal disorders [14]. Patients who have higher pain intensity, more widespread pain, and longer lasting pain report more sleep problems [15,16]. Studies regarding insomnia, obstructive sleep apnea (OSA), and experimental sleep deprivation and fragmentation have all suggested alterations at the metabolomics level. These include elevated levels of branched-chain amino acids (BCAAs) and altered glucose metabolism [17]. Obesity is another problem co-occurring with more severe pain [18]. It is associated with many metabolomic changes, such as elevated levels of BCAAs and aromatic amino acids (AAAs), and changes in acylcarnitines, fatty acids (such as phospholipids), and carbohydrates (such as glucose and mannose) [19].

In the present study, 110 polar metabolite serum markers covering 24 metabolite classes [20] were acquired from a cohort of patients analyzed previously for pain phenotype subgroup structure. We examined the associations of these markers with the identified subgroups (lower pain intensity and less interfering pain vs. higher pain intensity, more interfering pain, and more co-occurring problems) [13] in a data-driven approach using machine learning algorithms [21] and related feature selection techniques [22]. To further explore the complex reciprocal interactions between pain and co-occurring problems, we investigated metabolic pathways in relation to obesity and sleep problems, expecting to find alterations in, for example, amino acid metabolism [17,19]. Finally, we were interested whether these three analyses would suggest some common metabolomic markers or interacting mechanisms for those with more severe pain, and co-occurring obesity and sleep problems.

## 2. Results

**Figure 1:**
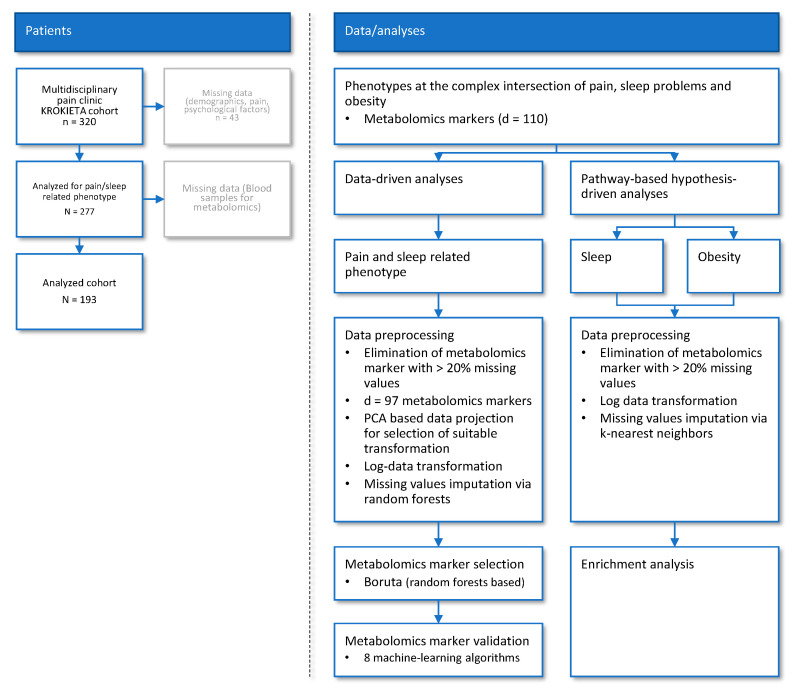
Flowchart showing number of patients and steps of data analysis. The data analysis followed two main lines: (i) A data-driven, unbiased approach to identify the most informative metabolomic markers for segregating patient subgroups in relation to the pain- and sleep-related phenotypes previously identified in the same cohort [13]; and (ii) a hypothesis-driven enrichment analysis examining metabolomic markers involved in sleep problems and obesity as main features of the patients’ clinical picture. The figure was created using Microsoft PowerPoint^®^ (Redmond, WA, USA) on Microsoft Windows 11 running in a virtual machine powered by VirtualBox 6.1 (Oracle Corporation, Austin, TX, USA).

Of the n = 320 patients with persistent pain treated in tertiary care, n = 277 patients had the necessary information about pain to be included in the previous analysis (Figure 1). This had established a patient subgroup structure based on relevant pain-related and other clinical symptoms [13]. Since in 84 of these patients the metabolomics had not been analyzed (due to patient non-compliance), the cohort analyzed comprised 71 men and 122 women. Descriptive statistical parameters relating to patient demographics, living situation, other pains, treatment experiences, comorbidities, and lifestyle are shown in Table 1. The raw data from the metabolomic markers are shown in Supplementary Figure S1.

### 2.1. Metabolomic Markers Informative for Pain Phenotype Assignment

Of the analyzed patients, 57 belonged to the subgroup characterized by lower pain intensity and less pain interference. Sex and age distributions were equal across the subgroups (sex: χ^2^ = 0.23101, df (degrees of freedom) = 1, *p* = 0.6308, age: t = −0.16011, df = 86.444, *p* = 0.8732); however, BMI was lower in the patients belonging to the group with lower pain intensity and less pain interference (26.3 ± 5.15 kg/m^2^) than in the other patients (29.2 ± 6.1 kg/m^2^; t = −3.7273, df = 121.11, *p* = 0.0002956).

**Figure 2:**
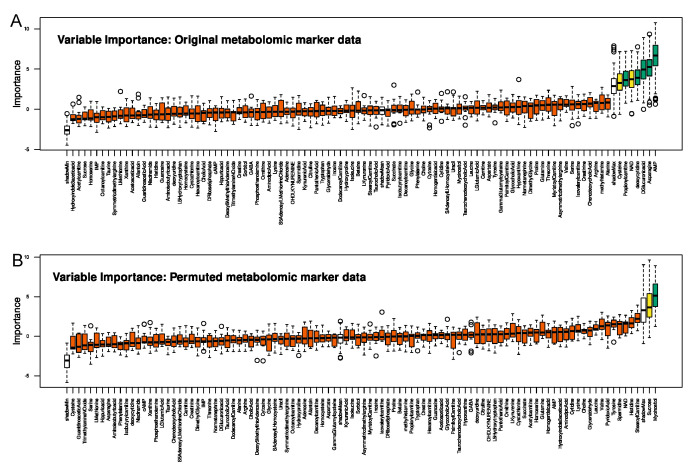
Identification and validation of metabolomic markers relevant for the assignment of patients to the correct pain phenotype subgroups. (**A**): Results of the variable selection procedure performed as random forest-based Boruta analysis, which assesses the measure of importance of a variable based on the decrease in classification accuracy due to random permutation of values in a 100-fold cross-validated approach. The importance measure is calculated separately for all trees in the forest that use the respective feature for classification. Then the mean value and the standard deviation of the loss of accuracy are calculated and the z-score is used in comparison to an external reference, the so-called “shadow” features (empty boxes), obtained by permuting the values of the original feature. Green and yellow boxes represent “confirmed” or tentatively significant features, respectively, i.e., features that contribute to the classification success and were selected for the validation analyses shown in the lower line of panels. The red boxes are confirmed as non-informative variables and excluded from further analysis. The boxes were constructed using the minimum, quartiles, median (solid line inside the box), and maximum of these values. The whiskers add 1.5 times the interquartile range (IQR) to the 75th percentile or subtract 1.5 times the IQR from the 25th percentile. The black circles indicate outliers from this interval. (**B**): Results of the Boruta feature selection analysis when instead of the original data, randomly permuted metabolic marker concentrations were used. The figure was created using the R software package (version 4.0.2 for Linux; https://CRAN.R-project.org/ [23]) and the R libraries “Boruta” (https://cran.r-project.org/package=Boruta [24]) and “ggplot2” (https://cran.r-project.org/package=ggplot2 [25]).

The Boruta feature selection analysis identified five metabolomic markers definitely important (AMP, asparagine, deoxycytidine, glucuronic acid, and propionylcarnitine), two others as tentatively important (cysteine and nicotinamide adenine dinucleotide, NAD), while 90 markers were classified as unimportant and excluded from further analyses (Figure 2). Of note, none of these markers was found when running the same algorithm on permuted metabolomic data. Because the five confirmed metabolomic markers were insufficient in subsequent validation to successfully train a random forest or other classifier, all seven above-mentioned markers were treated as important for the pain-related subgroup structure of this cohort. When training random forest classifiers with these markers (Table 2), balanced classification accuracy was better than random assignment, and AUC-ROC was 70% (66.3–72.2%). Balanced classification accuracy was also better than random assignment for the other machine-learning algorithms used to validate the selected set of metabolomic markers.

**Figure 3:**
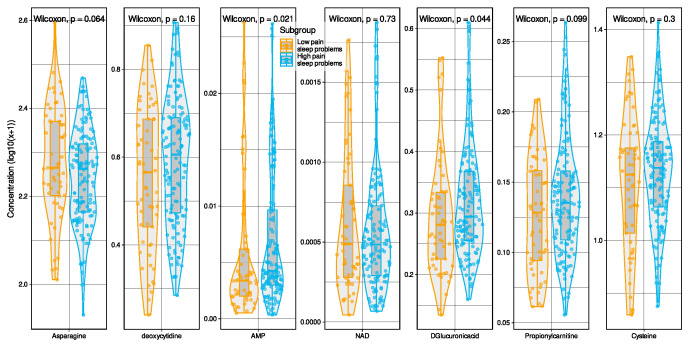
Raw data of the selected metabolomics features presented separately for the two pain and sleep-related subgroups. The transformed values (log10(x + 1)) are shown; for untransformed values of all metabolomic markers see Supplementary Figure S1. Individual data points are presented as dots on violin plots showing the probability density distribution of the variables, overlaid with box plots where the boxes were constructed using the minimum, quartiles, median (solid line inside the box), and maximum of these values. The whiskers add 1.5 times the interquartile range (IQR) to the 75th percentile or subtract 1.5 times the IQR from the 25th percentile. Statistical significances are shown at the top of each panel. The figure was created using the R software package (version 4.0.2 for Linux; https://CRAN.R-project.org/ [23]) and the R libraries “Boruta” (https://cran.r-project.org/package=Boruta [24]) and “ggplot2” (https://cran.r-project.org/package=ggplot2 [25]).

Of note, statistical comparisons of markers between patient subgroups using Wilcoxon–Mann–Whitney U tests (Figure 3) showed significant effects for two of the selected markers, AMP (W = 3057, *p* = 0.0208) and glucuronic acid (W = 3163, *p* = 0. 04415), both of which were lower in the low-pain subjects, while, for two others, group differences showed a tendency toward statistical significance (*p* < 0.1: asparagine, propionylcarnitine); however, none of these effects would pass a Bonferroni α correction.

### 2.2. Metabolomic Markers Relevant to Obesity and Sleep

**Figure 4:**
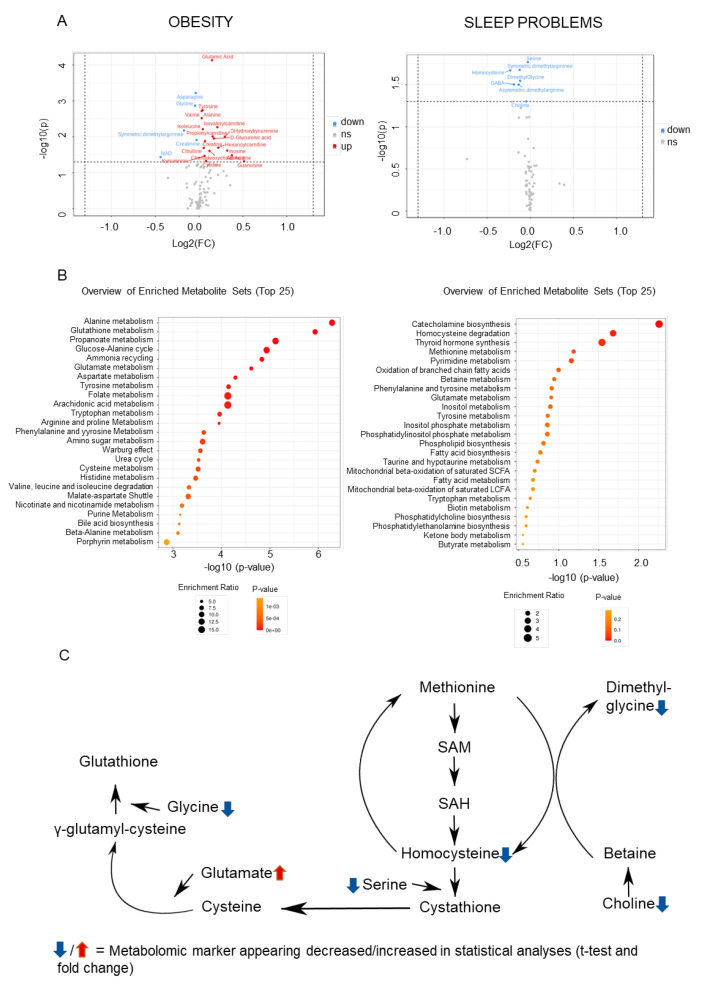
(**A**): Volcano plot showing the results for elucidating discerning markers between obese (BMI > 30) and non-obese patients, and those with recurring sleep problems, and those with normal sleep or only mild sleep problems. FC = 1, *p*-value < 0.05, BMI > 30/BMI < 30 and recuring/normal and mild sleep problems. FC = 1, *p*-value < 0.05 (**B**): Top 25 metabolic pathways that pathway enrichment analysis using SMPDB database suggested as having the most alterations, on the left in relation to obesity, and on the right in relation to sleep problems. The figure has been created using the MetaboAnalyst software (version 5.0, https://www.metaboanalyst.ca/home.xhtml [28]. (**C**): Mechanistic model illustrating how problems co-occurring with chronic pain may link at the metabolomic level. On the partial description of methionine metabolism (right), the blue arrows show the four metabolomic markers that are decreased in the recurring sleep problems subgroup in statistical analysis, suggesting downregulated methionine metabolism in this subgroup. Methionine metabolism is an important source of cysteine, needed for glutathione metabolism (left), and which appeared altered with obesity. The figure was created using Microsoft PowerPoint^®^ (Redmond, WA, USA) on Microsoft Windows 11.

Examination of the effects of obesity and sleep problems on metabolomics continued with univariate statistical analysis (*t*-test and fold change) and metabolite pathway analyses. The volcano plot analysis (Table 3), used to visualize the data, showed metabolomic markers with statistically significant differences when patients with obesity were compared to those without: 11 amino acids (glutamate, asparagine, glycine, tyrosine, valine, alanine, isoleucine, symmetric dimethylarginine, creatinine, creatine, citrulline), three acyl-carnitines (isovalerylcarnitine, propionylcarnitine, hexanoylcarnitine), two alkyl-phenylketones (hydroxykynurenine, kynurenine), sugar acid (glucuronic acid), three purine nucleosides (inosine, adenosine, guanosine), a bile acid (chenodeoxycholic acid), a (5′->5′)-dinucleotide (NAD), and a pyrimidine nucleoside (cytidine). In the pathway analysis (Figure 4), the top 10 enriched metabolic routes were related to amino acid metabolism and energy production, among others. Further detailed results are provided in Supplementary Table S1.

In the t-test analysis (Table 3), seven metabolomic markers differed statistically significantly when those having recurring sleep problems were compared to those sleeping normally or having only mild sleep problems: six amino acids (serine, symmetric dimethylarginine, homocysteine, dimethylglycine, GABA, asymmetric dimethylarginine) and choline. In pathway analysis (Figure 4), the top enriched metabolic routes related to phospholipid synthesis and methionine metabolism, among others.

### 2.3. Convergences in the Findings between the Machine-Learning Approach and Pathway Analyses

Contrasting the findings from the machine learning approach with pathway analyses it showed that four metabolites appeared both in the machine-learning-derived algorithm and in the top 25 metabolic pathways in relation to obesity: NAD (in 18 pathways), AMP (11), cysteine (4), and asparagine (2). For sleep problems, three of the same metabolites appeared in the machine-learning-derived algorithm and in the top 25 metabolic pathways: NAD (in 17 pathways), AMP (4), and cysteine (2). Therefore, markers appearing in all these analyses were AMP, NAD, and cysteine.

## 3. Discussion

This study sought first to elucidate metabolomic markers that were associated with having either a less severe pain phenotype (lower and less interfering pain) or a more difficult one (higher pain, more interfering pain, and more co-occurring problems). A data-driven machine-learning-based approach picked seven markers: AMP, asparagine, deoxycytidine, glucuronic acid, propionylcarnitine, cysteine, and NAD. Analysis of two common problems that associate with more difficult pain, i.e., obesity and sleep problems, implicated several metabolomic markers and pathways which may have an effect on pain. Further, three markers (NAD, AMP, and cysteine) appeared across the results from the machine learning and pathway analyses.

As described in the methods section, supervised machine learning classification algorithms were used for knowledge discovery rather than biomarker generation. The latter was not pursued for two reasons. First, the pain-related phenotype is a complex human phenotype that includes elements of both pain and sleep problems. This phenotype was based on a previous report of ours that suggested that sleep is a key factor in persistent pain [13]. The current study aimed to analyze metabolomic factors that may be involved in the process of pain chronification, not to identify biomarkers for e.g., diagnostic purposes. Second, understanding the involvement of metabolomic regulation in persistent pain is a new field of research and previous findings have so far not been able to formulate specific hypotheses-based metabolomics-derived data. Therefore, the present study aimed to gain further insight into the role of metabolomic regulation in human persistent pain with a special focus on the comorbidity of sleep.

Obesity is associated with more severe pain [18] and several mechanisms may explain this: e.g., heavier weight on joints and spine, depression, or low-grade chronic inflammatory state [29,30]. However, effects from differential metabolomics levels may emerge as well. As expected, alterations in amino acid metabolism pathways also appeared in this study [19]. A metabolomic profile, observed here too, of increased levels of BCAAs valine and isoleucine, and glutamate and alanine, has been hypothesized to reflect an overload of BCAA catabolism [31]. This may contribute to the development of glucose intolerance or affect neurotransmitter production, while increased levels of BCAAs may also be associated with increased inflammation, possibly leading to more pain [32,33]. Elevated glutamate, as excitatory neurotransmitter related to pain sensation, has been associated with greater pain in several studies [12]. Another interesting finding was that the metabolomic marker glucuronic acid appeared in both the machine-learning algorithm and the obesity-associated pathway analysis. Glucuronic acid has been shown to activate Toll-Like Receptor 4, leading to enhanced nociception possibly through the release of cytokines [34].

Sleep problems were associated with decreased levels in four metabolites (choline, homocysteine, dimethylglycine, and serine) in the methionine pathway. Experimental sleep deprivation in animals and humans reduces levels of cysteine [35] and homocysteine [36]. In response to simulated night shifts in humans, choline levels and those of two other metabolites in the methionine pathway decreased [37]. Homocysteine has been of much interest in research as elevated homocysteine levels have appeared as a risk factor for several diseases, including cardiovascular disease and dementia, and sleep problems have been proposed to play a part in this process [38]. However, sleep problems occur in various forms, and it may be that only obstructive sleep apnea (OSA) or severely reduced sleep durations (<5 h) link to elevated homocysteine [39,40]. OSA may induce more pain through chronic headaches [41] and metabolomic level alterations in OSA, such as disruptions in amino acid, fatty acid, carbohydrate, bilirubin, and xanthine metabolism, have been found [17,42]. Associations with metabolomics are often nonlinear and highly complex, as different pathways interact with one another. For pain relief, stimulating the methionine pathway has been studied in relation to chronic pancreatitis, hypothesizing that the effects would be mediated through reduced oxidative stress [43].

As pain, obesity, and sleep problems are showing to have reciprocal relationships, we were interested in the possible interactions across the metabolomic findings. One marker appearing in all three analyses was cysteine, a product of the methionine pathway, which is needed for glutathione synthesis [44]. Our results suggested that the methionine pathway is downregulated with sleep problems, while obesity associated with an altered glutathione pathway. If obesity affected glutathione metabolism through elevated glutamate or decreased glycine availability [45], could co-occurring sleep problems take the imbalance further through reduced cysteine availability? Glutathione plays an important role as an antioxidant defense and its deficit has been studied in relation to several diseases.

NAD, another common marker in the analyses, appeared in most pathways that both obesity and sleep problem analyses highlighted. Obesity is associated with decreased NAD levels and increased inflammatory cytokines are proposed as one possible mechanism for this [46]. Alterations of NAD levels may influence many processes as it has multiple functions, one of which is to do with the internal circadian clock, which then may play a part in sleep regulation [47].

Finally, increased AMP levels have been linked to obesity and diabetes [48]. AMP may also have direct effects on pain [49]. AMP is a hydrolysis product from ATP, a molecule which cells increasingly release in inflammation, tissue damage, or nerve injury. AMP is itself hydrolyzed to adenosine, which exhibits antihyperalgesic and antiallodynic effects. However, persistently elevated adenosine levels are associated with mechanical and thermal hypersensitivity, suggesting a possible role in chronic pain [50]. In our study, the serum level of AMP was higher in those with more pain and pain interference, suggesting that AMP hydrolysis might be affected in a variety of pain conditions, contributing to increased severe pain.

### Strengths and Limitations

This study analyzed metabolomics in pain patients with two complementary approaches. Data-driven methods may produce subgroup allocations that are more valid in the real world than those corresponding to some individual pain-related factor. Using machine learning to search for important combinations of metabolomic markers in pain subgroups and comparing these results to findings about two significant problems, obesity and sleep, among pain patients is a way to assess the validity of these results. Thus, combining different analysis strategies can be considered a strength of this study.

In addition, the presented set of metabolic markers derived from the data-driven part of the analysis has undergone several procedures to validate it and can therefore be considered as an internally validated result. In particular, (i) none of the markers emerged when feature selection was performed on permuted data. Moreover, (ii) algorithms other than random forests could be trained with these metabolic markers to assign cases to the correct phenotype group with a balanced accuracy that was better than guessing, whereas (iii) this was unsuccessful when training the algorithms with permuted data indicating that overfitting was unlikely. In addition, (iv) training the algorithms failed with markers that were significant in one-dimensional statistical analyses of group differences, suggesting a rejection of these markers found by simple statistics in favor of the markers found by the more complex approach pursued here, as discussed in the next section.

Only some, but not all, of the identified metabolomic markers that were instrumental for the assignment of pain phenotypes by different machine algorithms differed statistically significantly between the two phenotype groups (Figure 3). At first glance, this might call the present results into question. However, a rejection of the presented relevant metabolomic markers as predictors of pain phenotype due to lack of significance fails to recognize the high dimensionality of the data set and inadequately reduces it to a multiple unidimensional problem. In contrast, it has recently been shown that higher significance does not automatically mean stronger predictive power and variables with strong predictive power may be sometimes not significant [51]. As the authors of the report state, “the main difference between finding a subset of variables to be highly significant and finding them to be highly predictive is that the former involves making assumptions about the exact distribution of the variables but not knowing it, whereas the latter requires knowing the distribution of the variables in the classes under study”. Nevertheless, considering that some of the selected metabolomic markers were different from those that showed statistically significant differences between subgroups, we repeated the classification step of the data analysis using only the statistically significant metabolomic markers for algorithm training. In this case, classification performance fell back to the level of guessing.

The final set of metabolomic markers was found using a feature selection technique based on random forests, which is an established approach [24,52]. However, several additional analyses were performed to validate the final set of metabolomics markers. These included first classification algorithms other than random forests to ensure that the results were not due to properties of the random forests algorithm. In fact, SVM with the selected features provided even better classification performance than random forests. Second, training the algorithms with permuted metabolomics information resulted in their inability to assign patients to the correct cluster, demonstrating that the presented result was not due to overfitting or random selection.

Finally, as an alternative to the Boruta approach based on random forests, the method of least absolute shrinkage and selection operators (LASSO [53]) as a regression-based method was used as an alternative feature selection technique. LASSO identified alanine, GABA, serine, proline, betaine, valine, isoleucine, asparagine, creatine, hypoxanthine, glutamine, glutamate, citrulline, AMP, sorbitol, gamma glutamyl cysteine, guanosine, chenodeoxycholic acid, taurochenodeoxycholic acid, isobutyryl carnitine, and cysteine as informative. However, training random forests with this marker set resulted in poor balanced accuracy of only 51.5% (interquartile range of 49.3–53.1), and other algorithms did not provide support that this feature set as informative for subgroup assignment. Based on these observations, the present feature set seems to be sufficiently validated. Nevertheless, the small sample size is a limitation that must be considered when generalizing the present results.

## 4. Material and Methods

### 4.1. Subjects and Study Design

The cohort originally comprised n = 320 patients undergoing multidisciplinary therapy in tertiary pain care, enrolled between September 2013 and November 2016. The Coordinating Ethics Committee of Helsinki and Uusimaa Hospital District approved the study protocol (29.13.03.00/12). Informed written consent was obtained from all participants. The only exclusion criteria were active cancer or inability to answer questionnaires in Finnish. As described previously (see Table 1 in [13]), a total of d = 59 parameters in seven different categories, namely (i) pain phenotype-related features, (ii) pain etiology-related information, (iii) psychological parameters, (iv) demographic parameters, (v) lifestyle-related parameters, (vi) information about previous treatments, and (vii) information about comorbidities, had been acquired from the present cohort. Five of the pain-related parameters were used for pain-related clustering [13]; the other 54 parameters were not directly included in this cluster analysis but were used for later interpretation of the pain phenotype-derived clusters. Only the acquisition of information directly relevant to the present analysis, i.e., parameters related to pain, sleep, obesity, and metabolomics, are described below, while other details of the complete study have been described separately [13].

### 4.2. Pain-Related Phenotypes

As previously described [13,18], five pain-related parameters had been acquired from the patients, namely (i) pain intensity, (ii) activity pain interference, (iii) affective pain interference (assessed with the Brief Pain Inventory (BPI) [54]), (iv) the number of pain areas (from the pre-treatment health questionnaire, using an image of the human body on which the patient had marked areas with pain), and (v) the duration of pain.

Based on the patterns found with these parameters [13], a subgroup of 81 patients characterized by a relatively smaller number of pain areas and a lower level of affective pain interference was distinguished at the top level of the cluster dendrogram from the other patients. In interpreting this pain-related cluster structure with the 54 predominantly non-pain-related parameters mentioned above, using explainable artificial intelligence (XAI) type algorithms (i.e., which make cluster assignment transparent and understandable to non-informaticians (see [55] for another example of XAI in pain research)), sleep problems were consistently at the top of the rule hierarchy. This indicated that sleep provided the most relevant information for subgroup assignment, besides the pain-related parameters that had been used for cluster building. This provided the basis for identifying sleep as a central factor in chronic pain in the present cohort and provided a mixed pain- and sleep-related phenotype suitable for the aim of the present study to analyze the role of metabolomics at the interface of pain and sleep problems. Obesity is another major comorbidity with both pain and sleep problems and has clear metabolic implications [19] and was therefore chosen as one of the parameters to be studied in the metabolomic analyses. Those with obesity are more likely to suffer from various chronic pain conditions (for example chronic headaches, fibromyalgia, and joint pain) and population studies have suggested obesity as a risk factor for developing chronic pain. Research on potential biochemical mechanisms linking obesity with pain is rapidly growing [29].

### 4.3. Sleep and Obesity Parameters

Sleep problems were assessed using the previous criteria [56]. Briefly, subjective sleep difficulties were first queried using the sleep item from the 15D Health-Related Quality of Life (HRQoL) questionnaire [57]. In the 15D sleep item, respondents indicate whether they have normal sleep or mild, marked, great, or extreme sleep problems. Patients who reported normal sleep were classified under this category. Patients who reported at least marked sleep problems were assessed for recurrence of the problems, using the Basic Nordic Sleep Questionnaire (BNSQ) [58]. This a standardized questionnaire assessing sleep disturbances that asks about various symptoms in the past three months on a scale of 1 to 5 (1 = never or less than once a month; 2 = less than once a week; 3 = 1–2 nights a week; 4 = 3–5 nights a week; 5 = every night or almost every night). Patients were classified as having “recurrent sleep problems” if they reported at least one of the following problems: 1 = difficulty falling asleep at least three times per week; 2 = night-time awakenings at least three times per night, on at least three nights per week; 3 = feeling extremely tired after waking up in the morning at least three times per week. Additionally, daytime sleepiness also had to be reported at least three times per week. The remaining patients who neither reported sleeping normally nor met the criteria for recurrent sleep problems were classified as having “mild or infrequent sleep problems.” A patient was assigned as obese if the body mass index (BMI) was 30 or higher. The height and weight information used to calculate BMI were taken by a nurse while the patient visited the pain clinic for examination.

### 4.4. Serum Metabolomic Markers

Metabolomics were performed at the Finnish Institute of Molecular Medicine, using previously published methods [20]. Ten microliters of labelled internal standard mixture were added to 100 µL of biofluid sample and allowed to equilibrate. A total of 400 µL of extraction solvent (1% formic acid in acetonitrile) was added for protein precipitation. The samples were then centrifuged (14,000 rpm; 4 °C; 15 min); supernatants were collected and dispensed into phospholipid removal plate (Ostro^TM^, Waters Corporation, Milford, MA, USA), and then vacuum filtered (pressure differential 300–400 mbar; 2.5 min) on a Hamilton robot vacuum station. A total of 5 μL of filtered sample extract was injected into an ACQUITY UPLC system coupled to a Xevo^®^ TQ−S triple quadrupole mass spectrometer (Waters Corporation). Chromatographic separation was carried out with a 2.1 × 100 mm ACQUITY 1.7 µm BEH amide HILIC column (Waters Corporation) (temperature maintained at 45 °C). The total run time was 14.5 min including 2.5 min of equilibration step at a flow rate of 600 µL/min and subsequently, the gradient was created with mobile phase B (ACN/H_2_O, 90/10 (*v/v*), 20 mM ammonium formate, pH at 3) and mobile phase A (ACN/H_2_O, 50/50, ammonium formate, pH at 3) according to Nandania et al. [20]. About 5 µL of sample extract was injected with two cycles of washes, seal wash and partial loop. The detection system, a Xevo^®^ TQ−S MS (Waters Corporation), was operated with polarity switching electro spray ionization (ESI) having capillary voltage at 0.6 KV in both polarities. Throughout the experiment the following settings were used: the source temperature (120 °C), desolvation temperature (650 °C), high pure nitrogen and argon gas used as desolvation gas (600 L/hr) and collision gas (0.15 mL/min), respectively. Multiple reaction monitoring (MRM) acquisition mode was selected for quantification of metabolites (span time of 0.1 sec). MassLynx 4.1 software (Waters Corporation) was used for data acquisition, data handling, and instrument control. Data processing was done using TargetLynx software (Waters Corporation) and metabolites were quantified by using labeled internal standards and external calibration curves.

### 4.5. Data Analysis

#### 4.5.1. Data and Analysis Strategy

Data analysis was in two main parts (Figure 1). First, a data-science-based approach, using machine-learning-based feature selection methods [21,22] was pursued to identify metabolomic markers that could provide relevant information for assigning a patient to the correct pain phenotype subgroup. This approach was unbiased with respect to metabolomic markers or pathways potentially involved in the segregation of pain phenotype subgroups, analogously to the approach taken previously in a comparable “omics”-focused assessment [59]. Second, a metabolic pathway-based, hypothesis-driven approach, using metabolite set enrichment analysis (MSEA), was pursued to examine biologically meaningful patterns that are significantly enriched in the quantitative metabolomics data related to pathways relevant to sleep problems or obesity. The two lines of data analysis were performed independently by two researchers, resulting in differences in some details of the analyses, mainly due to the different software tools used and their default settings. The two parts were conducted independently to avoid mutual influence of the results, i.e., the characteristics selected in the first part were not considered in the second part and vice versa, which also allowed internal validation to a certain extent. Full details are provided below.

The data space in both lines of analysis had the form

$$D=\left\{\left(x_{i},y_{i}\right)|x_{i}\in \;X,\;y_{i}\in Y,\;i=1\ldots n\right\}$$

that consisted of a so-called input space X with the metabolomic markers collected from 193 patients. In addition, the so-called output space Y was included, which, in the first line of the analysis, consisted of class or subgroup information on the assignment of patients to the two pain phenotypes described above, and, in the second line of assignment, to the recurring sleep problems or obesity subgroups. Losses from the original 320 patients are due to (i) missing phenotypic information, resulting in only 277 patients being analyzed in the previous analysis [13], and (ii) metabolic information not available from 84 patients. Basic descriptive statistics were calculated, and group comparisons were performed using Wilcoxon-Mann-Whitney-U tests [60,61] or χ^2^ tests [62], with an α level set at 0.05 and corrected for multiple testing, according to the proposal of Bonferroni [63]. The main analyses were conducted independently by two researchers and are described below.

#### 4.5.2. Data-Driven Association of Metabolomic Markers and Pain Phenotypes

The programming work required for this part of the analyses was carried out in the R language [64] using the R software package [23], version 4.0.2 for Linux, which is available free of charge in the Comprehensive R Archive Network (CRAN) at https://CRAN.R−project.org/. Analyses were performed on an Intel Core i7−10510U (Intel Corporation, Santa Clara, CA, USA) notebook computer running Ubuntu Linux 20.04.1 LTS 64−bit (Canonical, London, UK)).

##### Data Preprocessing and Transformation

For the machine learning-based analyses, the data were preprocessed as follows. Subjects and variables with >20% missing values in the metabolomics information were eliminated, since for the machine-learning-based analyses, this had been defined as the limit for imputation, as used previously [13]. Therefore, only 97 metabolomic markers were included in these analyses. A transformation of the metabolomics data best suited for their association with the pre-established cluster structure (see above) was identified by means of PC−corr analysis [65]. This is an algorithm that complements principal component analysis (PCA) [66,67]. PC−corr attempts various transformations of the data for optimal segregation of the cohorts along a PC, which is evaluated by quantitative measures expressed as *p*-value, area under the receiver−operator characteristic (AUC−ROC), and area under the precision−recall curve (AUPR). Since the first principal component (PC) captures the largest possible variance in the data, optimum cluster segregation along this component was searched. This analysis was performed using an R-script provided with the description of the PC−corr analysis (pccorrv2.R, https://github.com/biomedical−cybernetics/PC−corr_net [65]). The analysis resulted in a recommendation for log transformation of the data as best suited to observe cluster segregation along the first PC. Missing values were replaced by non-parametric imputation by random forests [68,69], as implemented in the R library “missForest” (https://cran.r−project.org/package=missForest [70]).

##### Selection of Metabolomic Markers Informative for Pain−Phenotype Assignment

Metabolomic markers that provided relevant information for patient subgroup assignment were identified using supervised feature selection and machine learning. Feature selection [22] was implemented with the “Boruta” approach [24], which is based on the random-forests algorithm [68,69] as a generally well-performing classifier using a tree-based structure. The Boruta method provides a clear decision on whether a variable is important or not, derived from a 100-fold cross-validation approach and a statistical evaluation with *p*-values defaulting to 0.01 [24]. These calculations were performed with the R package “Boruta” (https://cran.r−project.org/package=Boruta [24]) with the default hyperparameter settings. It should be mentioned that it would be a problem to mix different types of feature selection algorithms. However, the analyses reported in Section 4.5.2 and Section 2.2 of this paper basically included only a one-dimensional feature analysis. This means that the relationships between feature dimensions, whether linear or more complex, are not considered. To further circumvent possible circularity, the Boruta method-based feature selection was performed again with permuted metabolome data, with the expectation that the validity of the selected features would be supported if they were not also selected from permuted data.

##### Validation of Metabolomic Markers Informative for Pain Phenotype Assignment

To assess whether the selected metabolomic markers indeed provided relevant information for subgroup assignment, various machine learning classification algorithms were trained to perform the task of assigning a patient to the correct subgroup, based on the information provided by the metabolomics data. This was performed with machine learning for knowledge discovery. The approach assumes that if a classifier can be trained to assign a patient to the correct class better than by guessing, the features (the metabolomic markers in the dataset needed by the classifier to accomplish this task) contain relevant information about the addressed class structure. In this way, the most informative markers can be identified. Thus, feature selection takes precedence over classifier performance whereas creating a powerful classifier to identify a biomarker is not the goal. This means that the analysis can be considered as successful if the class assignment is better than guessing and the variables needed for this assignment have been identified.

In order to assess whether the feature selection procedure identified a set of variables that provides enough information for class separation, several different machine-learning algorithms were trained with both full and reduced feature sets. A 100-fold cross validation scenario was run on disjoint training (2/3 of the cases) and test (1/3 of the cases) data subsets, randomly drawn from the original data set using Monte Carlo resampling [71] as implemented in the R library “sampling” (https://cran.r−project.org/package=sampling [72]). Classification performance was evaluated using standard measures comprising first balanced accuracy [73] as the main criterion, and then the AUC−ROC [74], sensitivity, specificity, precision, recall, positive and negative predictive value [75,76], and the F1 measure [77,78]. These calculations were performed with the R libraries “caret” (https://cran.r−project.org/package=caret [26]) and “pROC” (https://cran.r−project.org/package=pROC [27]).

To address possible circularity arising from feature selection and validation with random forests only, algorithms of supervised machine learning were selected to cover different types of classifiers, including methods previously applied to pain-related data [79] and included (i) random forests [68,69] as the algorithm used for feature selection, (ii) support vector machines (SVM) [80]), (iii) adaptive boosting [81], (iv) k-nearest neighbors (kNN) [82], (v) conditional inference trees (CTREE) [83], (vi) classification and regression trees (CART) [84], (vii) the hierarchical tree−based C5.0 classifier [85], and (viii) the non-hierarchical rules-generating partial decision trees classifier (PART) [86]. The R libraries used for these calculations comprised, in the above order of algorithms, “randomForest” (https://cran.r−project.org/package=randomForest [87]), “kernlab” (https://cran.r−project.org/package=kernlab [88]), “xgboost” (https://cran.r−project.org/package=xgboost [89]), “caret”, “party” (https://cran.r−project.org/package=party [83]), “rpart” (https://cran.r−project.org/package=rpart [90]), “C5.0” (https://CRAN.R−project.org/package=C50 [91], and “RWeka” (https://cran.r−project.org/package=RWeka [92]). Hyperparameters were tuned during grid searches (as performed previously [55]). For example, random forests were built with 500 trees and three features per tree, while the kNNs were used with the Euclidean distance and the value of k could be selected, based on an actual grid search performed on each run. SVM was executed with a radial-based Gaussian kernel, while CART was implemented with a minimum limit of five cases per split and a maximum tree depth of five decisions. To control possible overfitting, all machine-learning algorithms were additionally trained with randomly permuted features. The classifier trained with these data should not perform better than guessing, i.e., should give a balanced accuracy and an AUC−ROC equal or close to 50%. For examples of R code used for the best-performing classifiers random forest and SVM, see Box 1.

Box 1 **R code details for the best—performing classifiers random forest and SVM.**

SVM = {ActualClassifierObject <− ksvm(as.factor(Clusters) ~ ., data=TrainData, kernel=“rbfdot”, prob.model=TRUE, type = “nu−svc”)}

Defaults of the ksvm support vector machines method (for full details, see https://cran.r−project.org/web/packages/kernlab/kernlab.pdf):

ksvm(x, y = NULL, scaled = TRUE, type = NULL, kernel =“rbfdot”, kpar = “automatic”,C = 1, nu = 0.2, epsilon = 0.1, prob.model = FALSE, class.weights = NULL, cross = 0, fit = TRUE, cache = 40, tol = 0.001, shrinking = TRUE, ..., subset, na.action = na.omit)

RF = {ActualClassifierObject <− randomForest(as.factor(Clusters) ~ ., data = TrainData, mtry=3, ntree=500, na.action = na.roughfix)}

Defaults of the randomForest method (for full details, see https://cran.r−project.org/web/packages/randomForest/randomForest.pdf):

randomForest(x, y=NULL, xtest=NULL, ytest=NULL, ntree=500, mtry=if (!is.null(y) && !is.factor(y)) max(floor(ncol(x)/3), 1) else floor(sqrt(ncol(x))), weights=NULL, replace=TRUE, classwt=NULL, cutoff, strata, sampsize = if (replace) nrow(x) else ceiling(.632*nrow(x)), nodesize = if (!is.null(y) && !is.factor(y)) 5 else 1, maxnodes = NULL, importance=FALSE, localImp=FALSE, nPerm=1, proximity, oob.prox=proximity, norm.votes=TRUE, do.trace=FALSE, keep.forest=!is.null(y) && is.null(xtest), corr.bias=FALSE, keep.inbag=FALSE, ...)

#### 4.5.3. Pathway-Based Assessment of Metabolomic Markers Relevant to Sleep and Obesity

Pathway-based analyses were performed using prepackaged software tools available as a web-based comprehensive metabolomics data processing tool MetaboAnalyst (version 5.0, https://www.metaboanalyst.ca/home.xhtml; accessed on 1 September 2021, [28,93]. Metabolites were removed at a threshold of 20% missing values [94]. Log transformation and autoscaling were used to normalize the data. Missing values were imputed with k-nearest neighbors algorithm [82] based on similar samples. For univariate analysis, volcano plot analysis was performed using FC = 1 and *p*-value < 0.05. Pathway enrichment analyses were performed using the quantitative metabolite enrichment analysis (MSEA) algorithms in Metaboanalyst [95]. MSEA uses similar algorithms to those originally developed for Gene Set Enrichment Analysis (GSEA) [96]. KEGG metabolite IDs and a metabolic pathway-based SMPDB database (containing 99 metabolite sets to normal human metabolic pathways) were used. Metabolite sets containing at least two entries were used as cut off. Enrichment ratio was computed by hits/expected.

## 5. Conclusions

The results of this study suggest several metabolomic markers and pathways that may play a part in pain becoming more severe for some patients. Some effects may be more direct, such as our findings about AMP and the hypothesis that this might alter adenosine metabolism, leading to increased pain sensitivity. However, there may also be many indirect effects. For example, we found that NAD levels were altered in obesity: NAD appears in many metabolomic pathways and is associated with many functions, such as circadian rhythms, which may then influence sleep regulation. Then, as research has suggested, disturbed sleep may lead to greater pain through several processes. Our findings also raise the possibility that several problems co-occurring with pain may disturb metabolomic processes in an additive way: if sleep problems are associated with downregulating the methionine pathway and obesity with alterations in glutathione metabolism, what effects might occur when these two problems combine, given the known links between these pathways? Metabolomics is a promising new approach to gain understanding of processes in chronic pain, and clearly warrants further research.

## Figures and Tables

**Table 1:** Basic descriptive statistics of information in 53 parameters not primarily used for pain phenotype clustering [13], collected from the 193 patients included in the present analyses: patients’ demographics, living situation, other pains, medical treatment experiences and comorbidities, and lifestyle-related parameters. For ordinal and interval-scaled variables, medians with IQRs are reported; for categorical variables, the categories are shown with the counts of patients belonging to each. Raw non-imputed data are shown; counts < 193 indicate missing data for some patients.

Category	Variable	n	Median	Interquartile Range	Categories and n Per Category
Demographics	Age	193	48	38–56	-
	Sex	193	-	-	Men = 71 Women = 122
Living situation	No. of children	193	2	0–2	-
	Civil status	192	-	-	Married = 75 Registered relationship = 0 Cohabiting = 38 Unmarried = 49 Separated = 25 Widow = 5
	Education in years	188	13	11–15.13	-
	Type of work	193	-	-	Agriculture = 2 Manual work = 15 Office work = 94 Studying or at school = 9 Housewife = 2 Pensioner = 40 Unemployed = 31
	Household income	184	4	3–6	-
	Missed workdays within previous 12 mo	173	39	2–180	-
Pain related	No. of pain areas	193	3	2–5	-
	Duration of pain	193	-	-	<1 mo = 0 1–3 mo = 2 3–6 mo = 5 6–12 mo = 23 1–2 y = 30 >2 y = 123
	Pain intensity	193	6	5–6.75	-
	Affective pain interference	193	7	4.75–8.25	-
	Activity pain interference	193	6.67	5.67–8	-
	Any neuropathic pain	188	-	-	No = 117, yes = 71
	Low back pain	188	-	-	No = 132, yes = 56
	Musculoskeletal pain other than back pain	188	-	-	No = 145, yes = 43
	Facial pain	188	-	-	No = 178, yes = 10
	Abdominal pain	188	-	-	No = 181, yes = 7
	Complex regional pain syndrome	188	-	-	No = 177, yes = 11
	Headache	188	-	-	No = 184, 1 = 4
	Phantom pain	188	-	-	No = 188
	Fibromyalgia	188	-	-	No = 170, yes = 18
	Chronic pain syndrome	188	-	-	No = 184, yes = 4
	Other pain diagnosis	188	-	-	No = 168, yes = 20
Previous treatments	Negative treatment experiences	193	3	1–4	-
	Positive treatment experiences	193	4	2–6	-
	Physician visits within previous 12 mo	181	10	5–14	-
Comorbidities	Hypertension	192	-	-	No = 135, Yes = 57
	Heart failure	192	-	-	No = 187, Yes = 5
	Angina pectoris	192	-	-	No = 180, Yes = 12
	Diabetes	191	-	-	No = 175, Yes = 16
	Asthma	192	-	-	No = 160, Yes = 32
	Chronic obstructive pulmonary disease	192	-	-	No = 186, Yes = 6
	Rheumatoid arthritis	192	-	-	No = 190, Yes = 2
	Joint disease other than rheumatoid arthritis	192	-	-	No = 141, Yes = 51
	Low back pain	192	-	-	No = 91, Yes = 101
	Depression	190	-	-	No = 135, Yes = 55
	Psychiatric disorder other than depression	192	-	-	No = 181, Yes = 11
	Hypercholesterolemia ever in life	166	-	-	No = 94, Yes = 72
	Using cholesterol medication	168	-	-	No = 143, Yes = 25
	High blood pressure ever in life	190	-	-	No = 107, Yes = 83
	Blood pressure medication use ever in life	85	-	-	No = 28, Yes = 57
	Diabetes type	159	-	-	No = 130 No, but elevated blood sugar = 7 Yes, type 1 diabetes = 4 Yes, type 2 diabetes = 14 Yes, but don’t know type = 1 Yes, diabetes during pregnancy = 3
Lifestyle	Smoking currently	193	-	-	No = 118, yes = 75
	Exercise periods of >20 min per week	190	2	0–3	-
	Hours spent sitting per day	185	6	3.5–9.5	-
	Sleep problems index	190	17	14–20	-
	Nutritional index	135	1	1–2	-
	Drug abuse	135	0	0–0	No = 124 Has used = 10 Dependent = 1
	Alcohol consumption frequency	126	-	-	Never = 19 Once a month or less = 43 2–4 times a month = 40 2–3 times a week = 20 4 times a week or more = 4
	Body mass index	192	27.82	24.23–32.71	-
	Systolic blood pressure, mm Hg	193	135	124–150	-
	Diastolic blood pressure, mm Hg	193	86	80–94	-
	Waist circumference	192	95.25	84.5–106.25	-

**Table 2:** Performance measures for assigning subjects to the two clusters previously found in the pain patients [13], of which cluster #1 includes patients with comparatively few body areas in pain, low interference, little sleep disturbance, and low blood pressure. The performance of machine-learning-based random forest classifiers is given; for further algorithms, the selected main performance criterion (balanced accuracy) is shown in Supplementary Figure S2. Classification performance was measured (i) with the original data, (ii) with data sets designed to provide negative control by permutation of the original metabolomic parameters, and then with original or permuted data of those seven metabolomic markers found relevant to the patient subgrouping after feature selection (Figure 3). Results represent the medians (IQRs in parentheses) of the test performance measures from 1000 model runs using Monte Carlo resampling. The parameters correspond to the performance marker set implemented in the R libraries “caret” (https://cran.r-project.org/package=caret [26]) and “pROC” (https://cran.r-project.org/package=pROC [27]).

Parameter	Full Feature Set		Reduced Feature Set	
Feature set	Original	Permuted	Original	Permuted
Sensitivity, recall	0 (0–0)	0 (0–0)	31.6 (26.3–36.8)	10.5 (5.3–15.8)
Specificity	100 (97.8–100)	100 (100–100)	88.9 (84.4–91.1)	91.1 (86.7–93.3)
Positive predictive value, precision	0 (0–50)	50 (0–100)	53.6 (45.5–60)	33.3 (22.2–45.5)
Negative predictive value	70.3 (70.3–70.3)	70.3 (70.3–70.3)	75 (73.7–76.9)	70.5 (69.4–71.9)
F1	10 (9.5–10)	10 (10–10)	38.8 (33–45.2)	16.7 (14.3–25)
Balanced Accuracy	50 (49.9–50)	50 (50–50)	59.1 (57.1–62.9)	50.4 (47.8–53.5)
ROC-AUC	50.7 (46.5–56.1)	51.3 (46.7–55.1)	70 (66.3–75.2)	56.1 (49.3–61.6)

**Table 3:** Statistical analysis (fold change and t-test) used in volcano plot for elucidating discerning markers between obese (BMI > 30) and non-obese patients, and those with recurring sleep problems and those with normal sleep or only mild sleep problems.

Metabolomic Marker	FC	log2(FC)	Raw.Pval	−log10(p)
*Obesity*				
Glutamate	1.1076	0.14741	7.385 × 10^−5^	4.1317
Asparagine	0.97389	−0.038168	0.00060007	3.2218
Glycine	0.96871	−0.045858	0.0013494	2.8698
Tyrosine	1.0282	0.040139	0.0018034	2.7439
Valine	1.0209	0.029846	0.0019009	2.721
Alanine	1.0211	0.030172	0.0030191	2.5201
Isovalerylcarnitine	1.155	0.2079	0.0053701	2.27
Isoleucine	1.0301	0.042839	0.0061138	2.2137
Symmetric dimethylargininee	0.88753	−0.17213	0.0066633	2.1763
Propionylcarnitine	1.1127	0.15403	0.0097422	2.0113
Hydroxykynurenine	1.2256	0.29344	0.009839	2.007
Glucuronic acid	1.1245	0.16928	0.011138	1.9532
Creatinine	0.98053	−0.028359	0.012257	1.9116
Creatine	1.0483	0.068066	0.013068	1.8838
Hexanoylcarnitine	1.1638	0.21882	0.020064	1.6976
Citrulline	1.0376	0.053191	0.02039	1.6906
Inosine	1.2492	0.32101	0.02406	1.6187
Chenodeoxycholic Acid	1.0856	0.11852	0.024663	1.6079
Adenosine	1.2961	0.37413	0.032691	1.4856
Kynurenine	1.0443	0.062527	0.034	1.4685
NAD	0.73752	−0.43924	0.036641	1.436
Cytidine	1.0567	0.079523	0.047004	1.3279
Guanosine	1.4269	0.51284	0.047952	1.3192
*Sleep problems*				
Serine	0.98126	−0.027298	0.017081	1.7675
Symmetric dimethylarginine	0.91811	−0.12326	0.021126	1.6752
Homocysteine	0.85203	−0.23103	0.021403	1.6695
Dimethylglycine	0.9218	−0.11747	0.028466	1.5457
GABA	0.87712	−0.18915	0.03143	1.5027
Asymmetric dimethylarginine	0.91048	−0.1353	0.031587	1.5005
Choline	0.96778	−0.047256	0.049881	1.3021

FC = fold change.

## Data Availability

Data cannot be shared publicly because of ethical restrictions permitting only the release of group level data to protect patient privacy. Requests for subject level data may be made by submitting an application to the coordinating ethics committee of the Helsinki and Uusimaa Hospital District (please see https://www.hus.fi/en/researchers/Research_policy_and_procedure/Pages/default.aspx; secretary for the ethics committee tel. +358504286400, email eettiset.toimikunnat@hus.fi) and contacting the principal investigator for this study, Dr. Eija Kalso, via email (eija.kalso@helsinki.fi).

## Supplementary Materials

The following supporting information can be downloaded at: https://www.mdpi.com/article/10.3390/ijms23095085/s1.
