# Supplementary material for: Machine Learning and Pathway Analysis-Based Discovery of Metabolomic Markers Relating to Chronic Pain Phenotypes
Source: Int J Mol Sci. 2022 May 3;23(9):5085. doi: 10.3390/ijms23095085 (PMC9099732; doi:10.3390/ijms23095085)
Supplement: Supplementary file 1 [file ijms-23-05085-s001.zip › ijms-1668653-supplementary.pdf]

Supplementary information

Supplementary Figure S1: Boxplots of raw metabolomics data, without transformation and imputation.

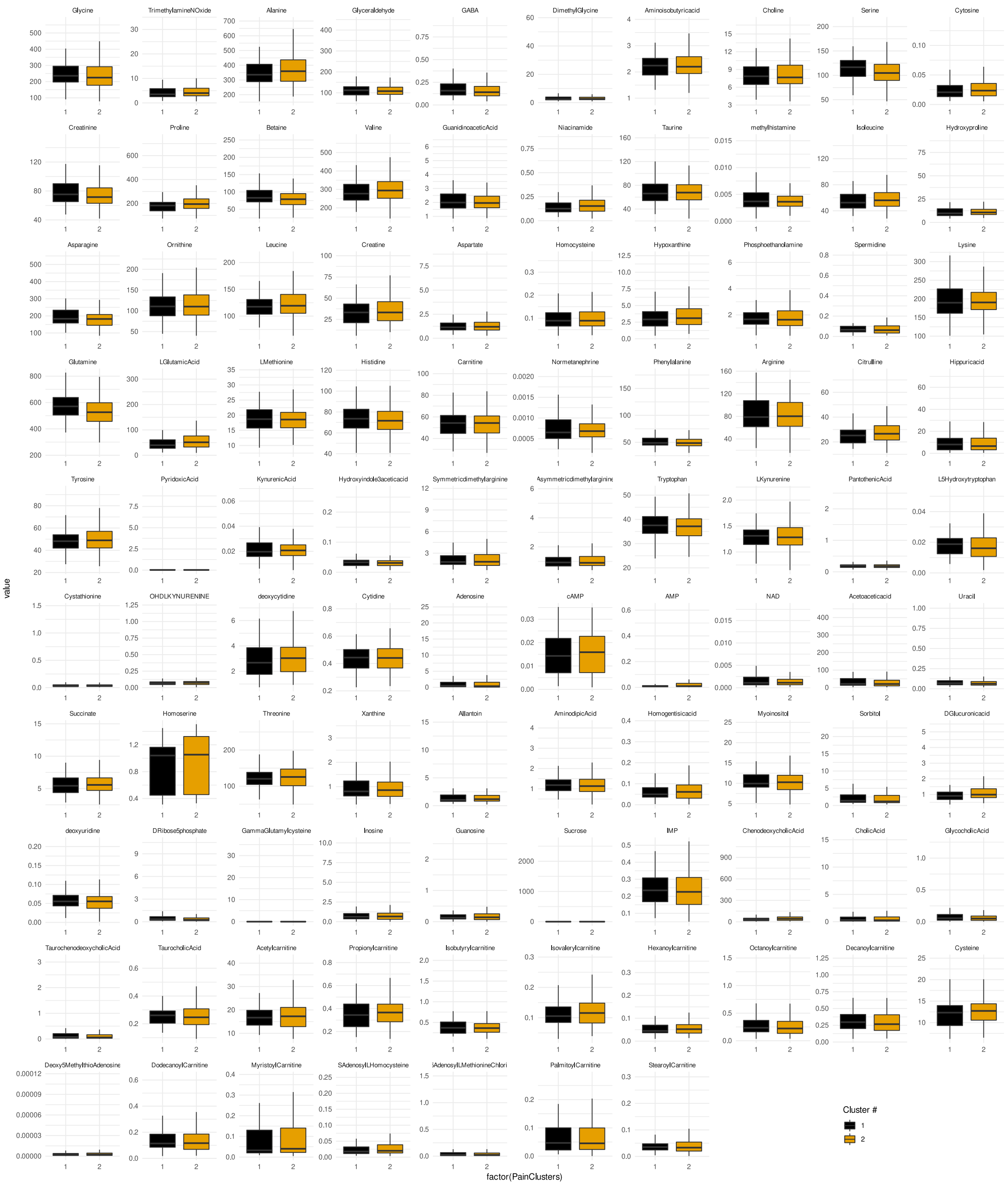

Supplementary Figure S2: Results of the variable validation procedure performed by training and testing eight different machine learning-based classification algorithms. Boxes show the balanced accuracy of the algorithms in assigning patients to pain- or sleep-related phenotype subgroups. **A:** Balanced classification accuracy when all d = 97 metabolomics markers were used to train the algorithms. The boxes were constructed using the minimum, quartiles, median (solid line inside the box) and maximum of these values. **B:** Balanced classification accuracy when only seven metabolomic markers selected by the Boruta method (panel A) were used for training the algorithms. **C:** Balanced classification accuracy when the algorithms were trained with the same metabolomic markers as in panel C but after random permutation. The figure was created using the R software package (version 4.0.2 for Linux; https://CRAN.R-project.org/) and the R libraries "Boruta" (https://cran.r-project.org/package=Boruta) and "ggplot2" (https://cran.r-project.org/package=ggplot2). RF: random forests; SVM: support vector machines; ADA: adaptive boosting; kNN: k-nearest neighbors; CTREE: conditional inference trees; RPart: classification and regression trees; PART: partial decision trees classifier. For details of hyperparameter settings, see the methods section.

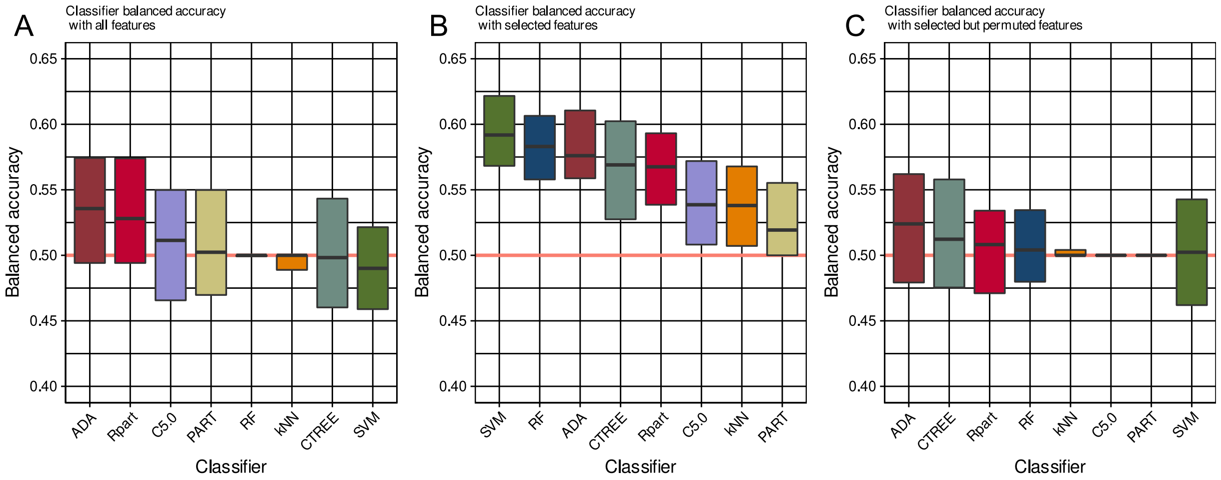

Supplementary Table S1: Enrichment sleep degradation. Holm-p = p-valued corrected for multiple testing according to the method proposed by Holm [1]), FDR = false discovery rate [2].

	Total Cmpd	Hits	Statistic Q	Expected Q	Raw p	Holm p	FDR
**Catecholamine Biosynthesis**	20	2	2.7279	0.5291	0.0055365	0.35433	0.35433
**Homocysteine Degradation**	9	2	2.0335	0.5291	0.020851	1	0.61261
**Thyroid hormone synthesis**	13	1	2.5195	0.5291	0.028716	1	0.61261
**Methionine Metabolism**	43	9	0.96779	0.5291	0.065093	1	0.68366
**Pyrimidine Metabolism**	59	3	1.2591	0.5291	0.069474	1	0.68366
**Oxidation of Branched Chain Fatty Acids**	26	4	1.0449	0.5291	0.10042	1	0.68366
**Betaine Metabolism**	21	6	0.92135	0.5291	0.11367	1	0.68366
**Phenylalanine and Tyrosine Metabolism**	28	4	0.98676	0.5291	0.12222	1	0.68366
**Glutamate Metabolism**	49	8	0.84747	0.5291	0.12406	1	0.68366
**Inositol Metabolism**	33	2	1.0921	0.5291	0.12713	1	0.68366
**Tyrosine Metabolism**	72	4	0.93306	0.5291	0.13726	1	0.68366
**Inositol Phosphate Metabolism**	26	1	1.1614	0.5291	0.13887	1	0.68366
**Phosphatidylinositol Phosphate Metabolism**	17	1	1.1614	0.5291	0.13887	1	0.68366
**Phospholipid Biosynthesis**	29	1	1.0724	0.5291	0.15507	1	0.70387
**Fatty Acid Biosynthesis**	35	1	1.0022	0.5291	0.16937	1	0.70387
**Taurine and Hypotaurine**	12	2	0.89666	0.5291	0.18405	1	0.70387

Metabolism							
Mitochondrial Beta-Oxidation of Short Chain Saturated Fatty Acids	27	2	0.84897	0.5291	0.19956	1	0.70387
Fatty acid Metabolism	43	1	0.83618	0.5291	0.20956	1	0.70387
Mitochondrial Beta-Oxidation of Long Chain Saturated Fatty Acids	28	1	0.83618	0.5291	0.20956	1	0.70387
Tryptophan Metabolism	60	4	0.74453	0.5291	0.22711	1	0.70387
Biotin Metabolism	8	1	0.7126	0.5291	0.24687	1	0.70387
Phosphatidylcholine Biosynthesis	14	2	0.71466	0.5291	0.25562	1	0.70387
Phosphatidylethanolamine Biosynthesis	12	2	0.71466	0.5291	0.25562	1	0.70387
Ketone Body Metabolism	13	2	0.66429	0.5291	0.28081	1	0.70387
Butyrate Metabolism	19	2	0.66429	0.5291	0.28081	1	0.70387
Bile Acid Biosynthesis	65	5	0.65677	0.5291	0.28595	1	0.70387
Phenylacetate Metabolism	9	1	0.5641	0.5291	0.30306	1	0.71836
Galactose Metabolism	38	3	0.5733	0.5291	0.35594	1	0.80278
Lysine Degradation	30	3	0.50972	0.5291	0.39313	1	0.80278
Aspartate Metabolism	35	7	0.54035	0.5291	0.40909	1	0.80278
Spermidine and Spermine Biosynthesis	18	2	0.46174	0.5291	0.41971	1	0.80278
Starch and Sucrose Metabolism	31	2	0.44128	0.5291	0.43081	1	0.80278
Pantothenate and CoA	21	2	0.43228	0.5291	0.43878	1	0.80278

Biosynthesis							
Beta Oxidation of Very Long Chain Fatty Acids	17	2	0.41466	0.5291	0.45419	1	0.80278
Selenoamino Acid Metabolism	28	2	0.41414	0.5291	0.4552	1	0.80278
Glycine and Serine Metabolism	59	13	0.51538	0.5291	0.46833	1	0.80278
Cysteine Metabolism	26	2	0.372	0.5291	0.48285	1	0.80278
Porphyrin Metabolism	40	1	0.25353	0.5291	0.49025	1	0.80278
Carnitine Synthesis	22	4	0.43744	0.5291	0.50463	1	0.80278
Ammonia Recycling	32	6	0.44771	0.5291	0.51558	1	0.80278
Methylhistidine Metabolism	4	1	0.2174	0.5291	0.52295	1	0.80278
Propanoate Metabolism	42	2	0.33079	0.5291	0.52683	1	0.80278
Nicotinate and Nicotinamide Metabolism	37	3	0.35827	0.5291	0.55446	1	0.81156
Glutathione Metabolism	21	4	0.37876	0.5291	0.56044	1	0.81156
Sphingolipid Metabolism	40	1	0.16134	0.5291	0.58216	1	0.81156
Valine, Leucine and Isoleucine Degradation	60	6	0.40933	0.5291	0.58447	1	0.81156
Fructose and Mannose Degradation	32	2	0.27559	0.5291	0.59599	1	0.81156
Amino Sugar Metabolism	33	2	0.22546	0.5291	0.63564	1	0.84752
Glucose-Alanine Cycle	13	2	0.21042	0.5291	0.65629	1	0.84769
Urea Cycle	29	7	0.36161	0.5291	0.67242	1	0.84769

**Folate Metabolism**	29	1	0.080468	0.5291	0.69764	1	0.84769
**Arachidonic Acid Metabolism**	69	1	0.080468	0.5291	0.69764	1	0.84769
**Alanine Metabolism**	17	3	0.21917	0.5291	0.72099	1	0.84769
**Glycerolipid Metabolism**	25	1	0.059519	0.5291	0.73829	1	0.84769
**Citric Acid Cycle**	32	1	0.051932	0.5291	0.75497	1	0.84769
**Mitochondrial Electron Transport Chain**	19	1	0.051932	0.5291	0.75497	1	0.84769
**Phytanic Acid Peroxisomal Oxidation**	26	1	0.051932	0.5291	0.75497	1	0.84769
**Histidine Metabolism**	43	2	0.13053	0.5291	0.77537	1	0.8524
**Warburg Effect**	58	3	0.17896	0.5291	0.78581	1	0.8524
**Purine Metabolism**	74	9	0.29951	0.5291	0.80091	1	0.8543
**Malate-Aspartate Shuttle**	10	2	0.09706	0.5291	0.81635	1	0.8565
**Threonine and 2-Oxobutanoate Degradation**	20	1	0.021711	0.5291	0.84009	1	0.86719
**Arginine and Proline Metabolism**	53	10	0.24	0.5291	0.91371	1	0.91533
**Beta-Alanine Metabolism**	34	4	0.12113	0.5291	0.91533	1	0.91533

R code extract for the classification step.

```
ClassifierChoice <- c("RF", "ADA", "SVM", "kNN", "CTREE", "Rpart", "C5.0", "PART")
nIter = 100
nCluster <- length(unique(ActualDataForClassification$Clusters))
if (nCluster > 2) {
  PerformanceActualClassifiersAll_ABCreduced <- data.frame(matrix(ncol = length(ClassifierChoice), nrow = nIter * nCluster))
} else { PerformanceActualClassifiersAll_ABCreduced <- data.frame(matrix(ncol = length(ClassifierChoice), nrow = nIter)) }
if (nCluster > 2) {
  PerformanceActualClassifiersAll <- data.frame(matrix(ncol = length(ClassifierChoice), nrow = nIter * nCluster))
} else { PerformanceActualClassifiersAll <- data.frame(matrix(ncol = length(ClassifierChoice), nrow = nIter)) }
if (nCluster > 2) {
  PerformanceActualClassifiersAll_ABCreducedPermuted <- data.frame(matrix(ncol = length(ClassifierChoice), nrow = nIter *
nCluster))
} else { PerformanceActualClassifiersAll_ABCreducedPermuted <- data.frame(matrix(ncol = length(ClassifierChoice), nrow =
nIter)) }
names(PerformanceActualClassifiersAll) <- ClassifierChoice
names(PerformanceActualClassifiersAll_ABCreduced) <- ClassifierChoice
names(PerformanceActualClassifiersAll_ABCreducedPermuted) <- ClassifierChoice
############# Feature selection
for (ii2 in 1:length(ClassifierChoice)) {
  ############# Classifier performance testing
  for (ii3 in 1:2) {
    ifelse(ii3 == 2, ActualDataReduced <- subset(ActualDataForClassification, select = c("Clusters",
ABCItemsBestcountBest)),
           ActualDataReduced <- ActualDataForClassification)
    if (ii3 == 2 & ii2 == 1) FinalFeatureData <- ActualDataReduced
    library(caTools)
    library(matrixStats)
    library(pROC)
    for (ii1 in 1:2) {
      PerformanceActualClassifier <- matrix(NA, nrow = 11, ncol = 0)
      rocAUC_ActualClassifier <- vector()
      for (i in 1:nIter) {
        set.seed(42 + i)
        sample <- sample.split(ActualDataReduced$Clusters, SplitRatio = .67)
        TrainData <- subset(ActualDataReduced, sample == TRUE)
        TestData <- subset(ActualDataReduced, sample == FALSE)
        if (ii1 == 2) {
          if (ii3 == 2) {
            TrainData[names(TrainData) %in% ABCItemsBestcountBest] <- apply(TrainData[names(TrainData) %in%
ABCItemsBestcountBest], 2, sample)
          } else {
            TrainData[2:ncol(TrainData)] <- apply(TrainData[2:ncol(TrainData)], 2, sample)
          }
        }
        MatrixTrain <- subset(TrainData, select = names(TrainData)[2:ncol(TrainData)])
        MatrixTest <- subset(TestData, select = names(TestData)[2:ncol(TestData)])
        UrsachenTrain <- TrainData[, 1]
        UrsachenTest <- TestData[, 1]
        switch(ClassifierChoice[ii2],
               ADA = {
          ActualClassifierObject <- rattle::xgboost(as.factor(Clusters) ~ ., data = TrainData,
                                                     max_depth = 5, eta = 0.25, num_parallel_tree = 5, nthread = 4,
                                                     nround = 50, verbose = F)
        },
               CTREE = {
          ActualClassifierObject <- ctree(as.factor(Clusters) ~ ., data = TrainData,
                                                  control = ctree_control(minbucket = 1, cores = 3, mincriterion = 0,
maxdepth = 5, minsplit = 5))
        },
               Rpart = {
          ActualClassifierObject <- rpart(as.factor(Clusters) ~ ., data = TrainData,
                                                  method = "class", xval = 1000, parms = list(split = "gini"), control
= rpart.control(cp = 0, maxdepth = 5, minsplit = 5))
        },
               C5.0 = { ActualClassifierObject <- C5.0(as.factor(Clusters) ~ ., data = TrainData, control =
C5.0Control(fuzzyThreshold = F, minCases = 5)) },
               PART = { ActualClassifierObject <- RWeka::PART(as.factor(Clusters) ~ ., data = TrainData) },
               RF = {
          ActualClassifierObject <- randomForest(as.factor(Clusters) ~ ., data = TrainData, mtry = 3, ntree = 500,
                                                      na.action = na.roughfix)
        },
               SVM = {
          ActualClassifierObject <- ksvm(as.factor(Clusters) ~ ., data = TrainData,
                                                 kernel = "rbfdot", prob.model = TRUE, type = "nu-svc")
        },
               kNN = {
          ctrl <- trainControl(method = "repeatedcv", repeats = 3) #,classProbs=TRUE,summaryFunction = twoClassSummary)
          ActualClassifierObject <- train(as.factor(Clusters) ~ ., data = TrainData,
                                          method = "knn", trControl = ctrl, preProcess = c("center", "scale"),
tuneLength = 10)
        },
        )
        if (ClassifierChoice[ii2] == "XXX") {
          Pred0 <- apply(ActualClassifierObject, 1, which.max)
        } else {
          Pred0 <- df_limePredict$predict
          Pred0 <- readr::parse_number(df_limePredict$predict)
        }
        Pred <- Pred0
        if (ClassifierChoice[ii2] == "ADA") {
          Pred <- round(scales::rescale(Pred0, to = c(1, 2)))
        } else {
          if (length(table(Pred0)) > nCluster | isTRUE(ncol(Pred0) > 1)) {
            if (ncol(Pred0) == nCluster) Pred <- as.vector(apply(Pred0, 1, which.max))
            else Pred <- round(scales::rescale(Pred0, to = c(1, 2)))
          }
        }
        cTab <- table(factor(Pred, levels = 1:nCluster), factor(TestData$Clusters, levels = 1:nCluster))
```

```
        ifelse(nCluster > 2, cMat <- t(caret::confusionMatrix(cTab)$byClass), cMat <- caret::confusionMatrix(cTab)$byClass)
        PerformanceActualClassifier <- cbind(PerformanceActualClassifier, cMat)
        if (ClassifierChoice[ii2] == "XXX") {
          Pred2 <- ActualClassifierObject
          colnames(Pred2) <- c(1, 2)
        } else {
          Pred2 <- as.matrix(subset(df_limePredict, select = level_temp))
          colnames(Pred2) <- c(1:nCluster)
          if (length(setdiff(names(table(Pred0)), as.character(unique(UrsachenTest)))) > 0) {
            if (ClassifierChoice[ii2] != "ADA") {
              if (ncol(Pred0) == nCluster) Pred2 <- Pred0
              else {
                Pred0resc <- scales::rescale(Pred0, to = c(0, 1))
                Pred2 <- data.frame(cbind(Pred0resc, Pred0resc))
                names(Pred2) <- c(1, 2)
                Pred2[, 2] <- 1 - Pred2[, 1]
              }
            }
          }
        }
        rocAUC_ActualClassifier <- append(rocAUC_ActualClassifier, multiclass.roc(TestData$Clusters, Pred2, quiet = T)$auc)
      }
      print(paste(ClassifierChoice[ii2], c("Original data", "Permuted data")[ii1]))
      print(paste("Data set", c("Full", "ABC reduced")[ii3]))
      print(rowQuantiles(PerformanceActualClassifier, probs = c(0.025, 0.25, 0.5, 0.75, 0.975), na.rm = T) * 100)
      print(quantile(rocAUC_ActualClassifier, probs = c(0.025, 0.25, 0.5, 0.75, 0.975), na.rm = T) * 100)
      if (ii1 == 1 & ii3 == 1) PerformanceActualClassifiersAll[, ii2] <- PerformanceActualClassifier[11,]
      if (ii1 == 1 & ii3 == 2) PerformanceActualClassifiersAll_ABCreduced[, ii2] <- PerformanceActualClassifier[11,]
      if (ii1 == 2 & ii3 == 2) PerformanceActualClassifiersAll_ABCreducedPermuted[, ii2] <- PerformanceActualClassifier[11,]
    }
  }
}
```

References

1. Holm, S. (1979). A Simple Sequentially Rejective Multiple Test Procedure. Scandinavian Journal of Statistics 6, 65-70.

2. Benjamini, Y., and Hochberg, Y. (1995). Controlling the false discovery rate - a practical and powerful approach to multiple testing. J. R. Stat. Soc. B. *57*.
